# Genetic and Diet-Induced Obesity Increased Intestinal Tumorigenesis in the Double Mutant Mouse Model Multiple Intestinal Neoplasia X Obese via Disturbed Glucose Regulation and Inflammation

**DOI:** 10.1155/2015/343479

**Published:** 2015-08-10

**Authors:** Ha Thi Ngo, Ragna Bogen Hetland, Unni Cecilie Nygaard, Inger-Lise Steffensen

**Affiliations:** Department of Food, Water and Cosmetics, Division of Environmental Medicine, Norwegian Institute of Public Health, P.O. Box 4404 Nydalen, 0403 Oslo, Norway

## Abstract

We have studied how spontaneous or carcinogen-induced intestinal tumorigenesis was affected by genetic or diet-induced obesity in C57BL/6J-*Apc* ^*Min*/+^ X C57BL/6J-*Lep* ^*ob*/+^ mice. Obesity was induced by the *obese* (*ob*) mutation in the *lep* gene coding for the hormone leptin, or by a 45% fat diet. The effects of obesity were examined on spontaneous intestinal tumors caused by the *multiple intestinal neoplasia* (*Min*) mutation in the *adenomatous polyposis coli* (*Apc*) gene and on tumors induced by the dietary carcinogen 2-amino-1-methyl-6-phenylimidazo[4,5-*b*]pyridine (PhIP). F1 ob/ob (homozygous mutated) mice had increased body weight (bw) and number of spontaneous and PhIP-induced small intestinal tumors (in *Apc* ^*Min*/+^ mice), versus ob/wt (heterozygous mutated) and wt/wt mice (homozygous wild-type). A 45% fat diet exacerbated bw and spontaneous tumor numbers versus 10% fat, but not PhIP-induced tumors. Except for bw, ob/wt and wt/wt were not significantly different. The obesity caused hyperglucosemia and insulinemia in ob/ob mice. A 45% fat diet further increased glucose, but not insulin. Inflammation was seen as increased TNF*α* levels in ob/ob mice. Thus the results implicate disturbed glucose regulation and inflammation as mechanisms involved in the association between obesity and intestinal tumorigenesis. Ob/ob mice had shorter lifespan than ob/wt and wt/wt mice.

## 1. Introduction

Obesity is defined as an excess accumulation of adipose tissue. The rate of obesity has more than doubled over the past 20 years in most OECD countries [1]. More than half of the adult population are overweight (with body mass index (BMI) 25–29.9 kg/m^2^) or obese (with BMI ≥ 30 kg/m^2^), and about 18% of both genders are obese. Rates of overweight and obesity among children are also increasing; average reported overweight rates (including obesity) increased from 13% in 2001-2002 to 15% in 2009-2010 for 15-year-olds (based on age- and gender-specific cut-off points for BMI) [1].

A parallel increase in overweight/obesity and many forms of cancer has been observed in most countries around the world in the past two to three decades. Cancer is now the second leading cause of mortality in the OECD countries [1]. In Norway, colon cancer is the second most prevalent cancer for women, after breast cancer, and the third most prevalent cancer for men, after prostate and lung cancer [2]. In the experiment described in this paper, we have examined how obesity may affect intestinal tumorigenesis.

Obesity alters the physiology of the whole organism, and therefore animal models are required to study the effects of increased adiposity. In addition, models that integrate lifestyle and genetic factors in a single model system provide a physiologically intact system valuable for studies of complex relationships. In the present work, we have studied the relationship between obesity and intestinal tumorigenesis in the double-mutant F1 offspring obtained by crossing a mouse model for intestinal tumorigenesis and a mouse model for obesity.

The well-established model for intestinal tumorigenesis, the C57BL/6J-*Apc* ^*Min/+*^ mouse, is heterozygous for the germline nonsense mutation* multiple intestinal neoplasia* (*Min*) in the tumor suppressor gene* adenomatous polyposis coli* (*Apc*) leading to a truncated nonfunctional APC protein, and therefore develops numerous spontaneous intestinal tumors [3, 4].* Apc* is a key component in the Wingless-related integration site (Wnt) signaling pathway [5, 6]. The* Min* mouse is a model for the inherited disorder familial adenomatous polyposis (FAP), as well as for sporadic colorectal cancer in humans [7, 8], and develops multiple adenomas in the small intestine and to a much lesser degree in the colon.

The* Min* mouse was crossed with the C57BL/6J-*Lep* ^*ob/+*^ mouse, heterozygous for the* obese* (*ob*) mutation in the* leptin* (*lep*) gene, which becomes obese when having a homozygous mutation (*ob/ob*) and therefore lacks functional leptin hormone [9–11]. In addition to being obese, the ob/ob mice are reported to exhibit hyperphagia, a transient diabetes-like syndrome of hyperglycemia, glucose intolerance, elevated plasma insulin, subfertility, impaired wound healing, and increased hormone production from pituitary and adrenal glands, and apparently they are hypometabolic and hypothermic [12]. Leptin regulates food intake and energy expenditure and has effects on immune functions, including inflammation, and reproduction [11, 13].

Obesity is caused by an imbalance between caloric intake and energy expenditure, which is influenced by both genetic and environmental factors. In this work, we have used two models for obesity. By studying obesity induced genetically in the* ob* mouse, we could examine the effect of obesity on intestinal tumorigenesis independent of diet. In addition, we studied environmentally-induced obesity, that is, diet-induced obesity (DIO), by giving the F1 mice either a 45% fat diet or a control 10% fat diet as adults (from weaning to termination).

In addition to the effects on spontaneous intestinal tumors caused by the inherited mutated* Apc* gene in the* Min/+* mice, the effect of obesity was also examined on tumors induced by the environmental (dietary) factor formed during cooking of meat and fish, the mutagenic, genotoxic and carcinogenic heterocyclic amine 2-amino-1-methyl-6-phenylimidazo[4,5-*b*]pyridine (PhIP) [14]. Previously, we have reported that PhIP increased intestinal tumorigenesis in adult C57BL/6J-*Min/+* mice [15] and that the* Min/+* mice were much more susceptible to PhIP if exposed neonatally [16, 17] than as young adults [15, 17].

In this work, we have studied obesity as an end point in itself, and as a factor impacting on intestinal tumorigenesis. The obesity was either caused genetically by the inherited* ob* mutation or environmentally by a 45% fat diet. We examined whether genetically-or diet-induced obesity increased spontaneous or carcinogen-induced intestinal tumorigenesis in the* Min* X* ob* mice. Furthermore, we examined two hypotheses for the relationship between obesity and intestinal tumorigenesis: disturbed blood glucose regulation and increased inflammation. In addition, we have examined the impact of heterozygous or homozygous* ob* mutation on long-term survival of mice with or without mutated* Apc* gene.

## 2. Materials and Methods

### 2.1. Mice

The* Min* (*multiple intestinal neoplasia*) pedigree was bred at The Norwegian Institute of Public Health, Oslo, Norway, by mating C57BL/6J-*Apc* ^*+/+*^ (B6J, wild-type) females with C57BL/6J-*Apc* ^*Min/+*^/J (stock 002020) males purchased from The Jackson Laboratory (Bar Harbor, ME, USA). To minimize the genetic drift away from the colony at the Jackson Laboratory, both females and males in the breeding stock at our institute were replaced regularly. Mice with the* obese* (*ob*) mutation in the* leptin* (*lep*) gene, B6.V-*Lep* ^*ob/+*^/J (stock 000632), were also purchased from The Jackson Laboratory.

### 2.2. Experimental Groups

In total, 48 experimental groups were included in this experiment: 6 genotypes (*Apc* ^*+/+*^ *X Lep* ^*wt*/*wt*^,* Apc* ^*+/+*^ *X Lep* ^*ob*/*wt*^,* Apc* ^*+/+*^ *X Lep* ^*ob/ob*^,* Apc* ^*Min/+*^ X *Lep* ^*wt*/*wt*^,* Apc* ^*Min/+*^ *X Lep* ^*ob*/*wt*^, or* Apc* ^*Min/+*^ *X Lep* ^*ob/ob*^) × 2 genders (females or males) × 2 treatments (0.9% NaCl or PhIP) × 2 diets (a 10% fat or a 45% fat diet). Homozygous mutant* Apc* ^*Min/Min*^ mice die during the embryo stages [18], whereas homozygous* Lep* ^*ob/ob*^ (*ob* ^*−/−*^) mice are viable [9, 11]. The* Min* mutation was propagated through males to avoid that the resulting intestinal adenomas and anemia might interfere with pregnancy in females [3]. Mice with leptin-deficiency (genetically induced obesity), that is, the* Lep* ^*ob/ob*^ mice, were used to study the effects of obesity separated from the influence of diet. Six genotype combinations were obtainable from crosses between* Min* mice and* ob* mice, via two generations. First,* Apc* ^*+/+*^ *X ob* ^*+/−*^ females and* Apc* ^*Min/+*^ X* ob* ^*+/+*^ males were crossed. Then the mice that were included in the experimental groups were produced by crossing of* Apc* ^*+/+*^ X* ob* ^*+/−*^ females and* Apc* ^*Min/+*^ *X ob* ^*+/−*^ males. To separate the wild-type (normal, nonmutated) allele of the* ob* gene from the wild-type allele of the* Apc* gene, throughout this paper the wild-type allele of the* ob* gene is designated “wt”, whereas the wild-type allele of* Apc* is designated “+.” The three* ob* genotypes obtained were wt/wt (homozygous wild-type), ob/wt (heterozygous mutated) and ob/ob (homozygous mutated), and the two* Apc* genotypes obtained were +/+ (homozygous wild-type) and Min/+ (heterozygous mutated).

Breeding of the experimental mice continued until approximately 11 mice per experimental group were obtained (number based on power analysis and experience with this model); however, to achieve this some groups ended up with higher numbers. The number of mice (*n*) per treatment group is given in the text and in figure legends and tables, for each end point.

### 2.3. Genotyping, Housing and Termination of the Mice

All* Min X ob* F1 offspring were genotyped for both* Min* and* ob* status by allele-specific polymerase chain reaction (PCR) analysis. DNA was extracted from ~2 mm^2^ samples obtained by ear puncture for identification of individual mice at weaning and kept on ice. The samples were suspended in 60 *μ*L TE-buffer with sodium dodecyl sulfate (SDS) (10 mM Tris pH 7.4, 0.1 mM EDTA pH 8.0, 0.05% SDS) and incubated at 95°C for 10 min. Then aliquots of 6 *μ*L of 10 mg/mL Proteinase K (Sigma-Aldrich Corp., St. Louis, MO, USA) were added and the samples incubated at 56°C overnight. Finally, the samples were incubated at 95°C for 10 min to inactivate the enzyme and stored at −20°C until PCR amplification. The PCR reactions for genotyping of* Apc* status were carried out as described previously [19]. For* ob* genotyping, the PCR reactions were carried out on an Eppendorf Mastercycler gradient (Eppendorf AG, Hamburg, Germany) as follows. Genomic DNA (5 *μ*L of 1 : 100 dilution of isolated DNA) was amplified in a 10 *μ*L reaction volume per sample, which contained final primer concentrations of 0.9 *μ*M of each of the following primers: oIMR1151 (5′-TGT CCA AGA TGG ACC AGA CTC-3′) and oIMR1152 (5′-ACT GGT CTG AGG CAG GGA GCA-3′), purchased from Eurogentec s.a. (Seraing, Belgium), 0.2 *μ*M of dCTP, dGTP, dTTP, and dATP (Promega Corp., Madison, WI, USA), 1x DyNazyme II Hot Start Reaction buffer (15 mM Tris-HCl (pH 8.2), 30 mM KCl, 2.5 mM Mg^2+^, 5 mM (NH_4_)SO_4_ and 0.02% bovine serum albumin (BSA)), and 0.008 U/*μ*L DyNazyme II Hot Start DNA polymerase (both from Thermo Fischer Scientific Inc., Waltham, MA, USA). The amplification conditions were 10 min at 94°C before 35 cycles at 94°C for 15 sec, 64°C for 30 sec and 72°C for 23 sec, followed by a final extension at 72°C for 5 min. After the PCR amplification, 10 *μ*L PCR products and 5 *μ*L restriction enzyme mix, which in the final concentration of 15 *μ*L volume contained 0.56x buffer D, 0.06 mg/mL BSA and 0.23 U Dde I enzyme (Promega Corp.), were incubated for 12 h. The PCR products were visualized by electrophoresis through a 2.2% agarose gel (Lonza FlashGel system, Lonza, Basel, Switzerland). The wild-type (wt/wt) mice were identified as having a 155 bp PCR product, the heterozygous mutated (ob/wt) mice with the 155 bp, a 100 bp and a 55 bp PCR product, and the homozygous mutated (ob/ob) mice as having a 100 bp and a 55 bp PCR product. The reagents were purchased from Sigma-Aldrich Corp. (St. Louis, MO, USA), Fluka (Buchs SG, Switzerland) and Promega Corp. (Madison, WI, USA), if not stated otherwise.

The littermates of the same gender were housed up to 5 mice per cage in 100% PET plastic disposable cages on Nestpak Aspen 4HK bedding (Datesand Ltd., Manchester, UK) in air flow IVC racks (Innovive Inc., San Diego, CA, USA). The room had 12-h light/dark cycle and controlled humidity (55 ± 5%) and temperature (20–24°C). Water and feed were given* ad libitum* to all mice.

The mice in the experimental groups were terminated at 11 weeks of age, before onset of noticeable anemia caused by their tumors. Blood was sampled by cardiac puncture under anesthesia with ZRF cocktail (containing 3.3 mg zolazepam, 3.3 mg tiletamine, 0.5 mg xylazine and 2.6 *μ*g fentanyl per mL 0.9% NaCl) into Microvette Lithium Heparin tubes for plasma from Sarstedt AS (Ski, Norway) for cytokine and hormone analyses. Thereafter, the mice were sacrificed by cervical dislocation.

### 2.4. Ethics Statement

The study was performed in strict accordance with the laws and regulations for animal experiments in Norway. The protocols were approved by the Norwegian Animal Research Authority (NARA) (permit numbers 1357 and 4856). Cardiac puncture and cervical dislocation were performed under ZRF cocktail anesthesia, before dissection of organs after death. Every effort was made to minimize suffering.

### 2.5. Dietary Carcinogen

2-Amino-1-methyl-6-phenylimidazo[4,5*b*]pyridine hydrochloride (PhIP-HCl) (CAS no. 105650-23-5), Cat. no. 163-15951, of >99% purity was purchased from Wako Chemicals GmbH (Neuss, Germany). PhIP-HCl was dissolved in distilled water, and the pH was adjusted to approximately 4.0.

### 2.6. Breeding and Experimental Diets

The breeding pairs, as well as their offspring until weaning at three weeks of age, were fed a breeding diet, 2018 Teklad Global 18% Protein Rodent Diet from Harlan Industries Inc. (Indianapolis, IN, USA). The mice bred for obtaining breeding pairs, not included in the experimental groups, were given a standard maintenance diet, SDS RM1 (E) from SDS Special Diets Services (Essex, UK), after weaning.

In addition to genetically-induced obesity caused by the* ob* mutation, we also studied the effects of diet-induced obesity (DIO), more relevant to the obese condition in most humans. Approximately every other litter of mice included in the experimental groups was given either a 45% fat diet or a 10% fat diet from weaning and until termination at 11 weeks. In order to make sure the effects observed were due to higher fat at the expense of carbohydrate only, and not decreases in proteins, vitamins or minerals, diets were chosen that added fat as % Atwater fuel energy (AFE) with isocaloric exchange with carbohydrate. The high fat diet used was 45% AFE fat diet, code 824053 (45% kcal from fat, 20% kcal from proteins, 35% from carbohydrate, 4.54 kcal AFE/g), for comparison with the normal fat diet 10% AFE fat diet, code 824050 (10% kcal from fat, 20% kcal from proteins, 70% from carbohydrate, 3.68 kcal AFE/g), both from SDS Special Diets Services.

### 2.7. Feed and Water Intake

Since the 45% fat diet crumbled easily it was not possible to determine the feed intake in a reliable way by weighing the feed in and out of the cages. Instead, feed and water intake were recorded in a selection of mice from each treatment group kept in metabolic cages for 24 h at age 9–11 weeks, after getting accustomed to the cages the day before. Urine volume was measured and urine and feces were collected for later use not reported in this paper.

### 2.8. Obesity Determined as Area under the Curve (AUC), Terminal Body Weight and Body Mass Index (BMI)

Body weight was recorded weekly from weaning at 3 weeks of age to termination at 11 weeks of age for all* Apc* ^*Min/+*^ and* Apc* ^*+/+*^ mice. At termination, nasoanal lengths were recorded for calculation of body mass index (BMI), defined as body weight divided by the nasoanal length squared (in g/cm^2^). Since different indicators of obesity might be more or less suited for evaluation of its relationship with end points such as intestinal tumorigenesis, we evaluated the body weight data in three different ways. The body weight data were analysed as area under the curve (AUC) for body weight recorded from 3 to 11 weeks of age, calculated using the macro in SigmaPlot 12.3 (Systat Software Inc., San Jose, CA, USA), which integrates the area under the curve using the trapezoidal rule. In addition, the body weight data were evaluated as terminal body weight and terminal BMI at 11 weeks (data not shown).

### 2.9. Induction and Scoring of Intestinal Tumors

F1 pups from* Min X ob* crosses were given a single subcutaneous (s.c.) injection of 25 mg/kg bw PhIP on day 3–6 after birth, for evaluation of effects of obesity on carcinogen-induced tumors. Separate litters were given a s.c. injection of 0.9% NaCl as vehicle controls for scoring of spontaneous tumors only.

The colon and small intestine were removed separately, rinsed in ice-cold phosphate-buffered saline (PBS) and slit open along the longitudinal axis. Intestinal tissues were then spread flat between sheets of filter paper and fixed for at least 48 h in 10% neutral buffered formalin prior to staining with 0.2% methylene blue (Sigma-Aldrich Norway AS). Number, diameter, and localization of tumors in small intestine and colon were scored by transillumination in an inverse light microscope at a magnification of ×20. The scoring was done in order of consecutive mouse numbers unaware of their treatment. Diameters of tumors were scored with an eyepiece graticule. Tumor position along the intestines was registered in cm from the stomach. For each experimental group, incidence of tumors (number of mice with tumors/number of mice in the group), tumor number (mean number of tumors/mouse ± SD) and tumor diameter in mm (mean of all tumors in all mice in the group ± SD) were calculated, for small intestine and colon separately. In addition, size of the tumors was illustrated by curves of distributions of tumor size classes (of 0.25 mm tumor diameter intervals) calculated as mean number of tumors in each tumor size class for each treatment group. In the C57BL/6J strain, the small intestine is the main target organ for tumorigenesis caused by the* Min* mutation as well as by PhIP, and very few tumors were found in the colon. Therefore, the colonic tumor data are not shown.

### 2.10. Glucose Tolerance Test (GTT) and Nonfasted Blood Glucose Measurements

A glucose tolerance test (GTT) was performed on a selection of mice from all treatment groups at age 6 weeks. These mice were fasted for 6 h (8-9 a.m. to 2-3 p.m.) and were given an intraperitoneal (i.p.) injection of 2 g/kg bw D-(+)-glucose (Sigma-Aldrich Norway AS, Oslo, Norway). Glucose in blood obtained by puncture of the saphenous vein was measured with a glucometer (FreeStyle Freedom Lite (Abbott Diabetes Care Inc., Alameda, CA, USA) 5 min before and 15, 30, 60 and 120 min after the glucose injection. The area under the glucose tolerance curve (AUC) was calculated from −5 to 120 min, using the macro in SigmaPlot 12.3. When readings were >27.8 mmol/L, displaying HIGH in the glucometer, this value was used in the data analysis. This was found for 5, 2 and 48% of the samples in one or several time points in wt/wt, ob/wt and ob/ob mice, respectively. No samples had glucose readings below 1.1 mmol/L and showing LOW in the glucometer. Nonfasted blood glucose levels were also measured in all mice at both age 6 and 11 weeks.

### 2.11. Urine Glucose Measurements

In cases with raised blood glucose concentrations, for instance, in diabetes, glucose may be found in the urine when its concentration in plasma exceeds the renal threshold. In some mice showing high values of blood glucose, urine glucose was also measured by urinalysis dipsticks Siemens Multistix 8 SG purchased from Siemens Healthcare Diagnostics Inc. (Tarrytown, NY, USA).

### 2.12. Insulin and Insulin-Like Growth Factor (IGF-1) Analyses

Insulin and insulin-like growth factor 1 (IGF-1) were measured in plasma obtained from the mice at sacrifice. ELISA kits from MyBioSource Inc. (San Diego, CA, USA) were used according to the manufacturer's instructions. Optical density (OD) was measured at 450 nm on a BioTek microplate reader (BioTek Instruments Inc., Winooski, VT, USA). Concentrations were calculated from standard curves on each plate. All samples were diluted 1 : 5 in PBS, pH 7.1. The limit of detection was 0.1 ng/mL for both insulin and IGF-1.

### 2.13. Cytokine Analyses

The proinflammatory cytokines interleukin-1*β* (IL-1*β*), interleukin-6 (IL-6) and tumor necrosis factor *α* (TNF*α*) were measured in plasma obtained from the mice at sacrifice. Bead-based immunoassays BD Cytometric Bead Array (CBA), from BD Biosciences, San Jose, CA, USA, were used according to the manufacturer's instructions. Data were collected on a BD LSRII flow cytometer (BD Biosciences) and analysed by use of FCAP Array software 3.0 (BD Biosciences). All samples were diluted 1 : 2 in Assay Diluent included in the CBA kit. The limits of detection were 1.9, 1.4 and 2.8 pg/mL, for IL-1*β*, IL-6 and TNF*α*, respectively.

### 2.14. Long-Term Survival of Untreated ob Mice

The impact of the* ob* genotype on life-span was examined in groups of untreated* Apc* ^*Min/+*^ and* Apc* ^*+/+*^ mice, of all three* ob* subgroups, ob/ob, ob/wt and wt/wt, and both genders. The mice were kept under regular observation and euthanized by cervical dislocation when deterioration in general health or quality of life were reached, as judged by clinical observations, body condition and symptoms such as weight loss, dehydration or hunched posture. Survival of each genotype of mice (in days) was depicted as decreasing % of surviving mice compared with the number of mice present at the start of the experiment.

### 2.15. Statistical Analyses

The data for each end point (such as body weight, blood glucose levels and tumor numbers) was evaluated on several levels; first on all mice and then further on several strata; age,* Apc* genotype, 0.9% NaCl or PhIP treatment, gender,* ob* genotype and % fat diet. The results are reported down to the most specific combination of these parameters for which statistically significant differences were found. Because of this complexity, statistical comparisons cannot be shown in a meaningful way on the figures or in Table 1, which give the numerical data. However, the *P* values of all comparisons are given in the text. All data were presented as mean ± SD, except for the cytokine and insulin data where median and single values were shown, and were analysed using SigmaPlot 12.3. Student's *t*-test or Mann-Whitney Rank sum test for nonparametric data was used for testing of the survival data. The incidence of colonic tumors was analysed by Fischer exact test (two-tailed probability). Simple and multiple linear regression were used to examine the associations between body weight, glucose, insulin and cytokine levels and number or diameter of small intestinal tumors. For all other data, analysis of variance (ANOVA) was used with an appropriate multiple comparison procedure. When testing the influence of a single factor, one-way ANOVA with the Holm-Sidak test for multiple comparisons was used for parametric data or the Kruskal-Wallis ANOVA on ranks with Dunn's test for multiple comparisons was used for nonparametric data. When testing the influences of two or three factors together the data were analysed by two- or three-way ANOVA, respectively, with the Holm-Sidak test for multiple comparisons. A *P* value of < 0.05 was considered statistically significant.

## 3. Results

### 3.1. Feed and Water Intake

The feed and water intake were recorded in mice kept in metabolic cages for 24 h. When the feed and water intake data were evaluated as g feed or mL water/g bw/day, there were no significant differences in feed or water intake between* Apc* ^*Min/+*^ and* Apc* ^*+/+*^ mice, or between 0.9% NaCl- or PhIP-treated mice. Therefore, the data were pooled for* Apc* genotype and 0.9% NaCl or PhIP treatment, and stratified only for gender,* ob* genotype and % fat diet (Table 1).

The feed intake per g bw was higher in females than in males, based on all mice (*P* < 0.001), and in the subgroups ob/wt (*P* = 0.009) and wt/wt (*P* < 0.001) mice, but not in ob/ob mice. A higher feed intake per g bw in females than in males was also found in a previous experiment [19].

Mice fed a 10% fat diet had significantly higher feed intake than mice fed a 45% fat diet (*P* < 0.001). This was also seen in the subgroups ob/ob, ob/wt and wt/wt mice, exposed to either 0.9% NaCl or PhIP (*P* < 0.001, for all comparisons).

The ob/ob mice had significantly lower feed intake per g bw compared with ob/wt and wt/wt mice (*P* < 0.001, for both comparisons), whereas ob/wt and wt/wt mice were not significantly different. In females separately, the results were the same as for all mice, whereas in males separately, the differences between the three* ob* genotypes did not reach significance. The feed intake per mouse was significantly higher in ob/ob mice than in ob/wt and wt/wt mice, but because of the extreme body weight of the ob/ob mice, the intake was lower on a body weight basis.

When the feed intake was compared as intake of kcal/g bw during 24 h, the results were essentially the same as for g feed/g bw (Table 1).

The water intake per g bw was higher in females than in males, based on all mice (*P* < 0.001), and in the subgroups of mice on a 10% fat diet and on a 45% fat diet (*P* < 0.001 and *P* = 0.012, resp.), and in the subgroup of ob/ob mice (*P* = 0.003) (Table 1).

Mice fed a 10% fat diet had significantly higher water intake per g bw than mice fed a 45% fat diet (*P* < 0.001).

The ob/ob mice had significantly higher water intake per g bw compared with ob/wt and wt/wt mice (*P* = 0.010 and *P* = 0.009, resp.), whereas ob/wt and wt/wt mice were not significantly different. On the individual level, we observed that many, but not all, of the ob/ob mice had a much higher water intake as well as higher urine output than ob/wt and wt/wt mice, especially mice with blood glucose above normal levels. This condition appeared to be transient.

### 3.2. Obesity Determined as Area under the Curve (AUC), Terminal Body Weight and Body Mass Index (BMI)

**Figure 1:**
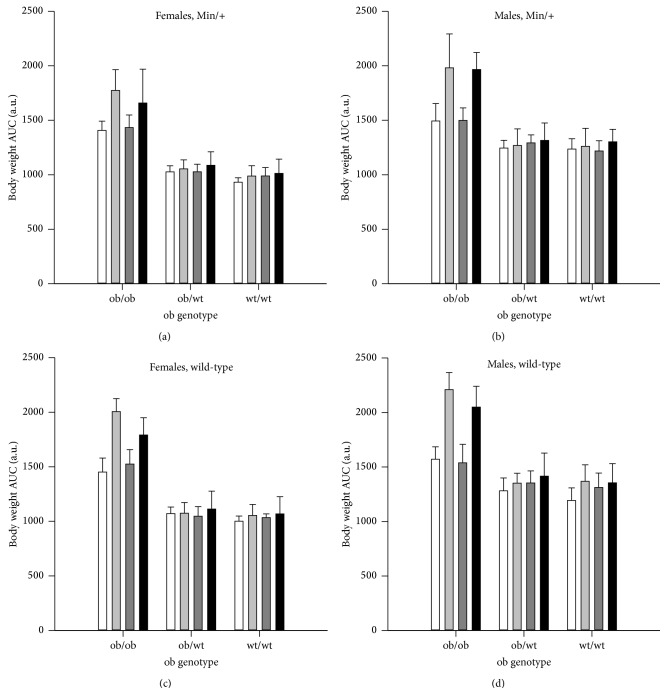
Body weight as area under the curve (AUC). Body weight was recorded weekly from weaning at week 3 until termination at week 11 and is presented for* Apc* ^*Min/+*^ (a) females and (b) males, and* Apc* ^*+/+*^ (c) females and (d) males (mean ± SD). The mice were exposed to 0.9% NaCl and given a 10% fat diet (white columns) or 0.9% NaCl and a 45% fat diet (light grey columns), or they were exposed to PhIP and given a 10% fat diet (dark grey columns) or PhIP and a 45% fat diet (black columns). *n* = 10–18 mice. a.u. = arbitrary units.

Body weight was compared between the treatment groups (*n* = 10–18) by calculating the area under the curve (AUC) for the body weight development from weaning at week 3 to termination at week 11 (Figure 1). For this end point, data from both* Apc* ^*Min/+*^ and* Apc* ^*+/+*^ were evaluated. The* Apc* ^*Min/+*^ mice were lighter than the* Apc* ^*+/+*^ mice (*P* < 0.001). There were no differences between mice treated with 0.9% NaCl or PhIP, based on all mice and in the subgroups ob/ob, ob/wt, and wt/wt mice. AUC was higher for males than females based on all mice (*P* < 0.001), in the subgroups ob/ob, ob/wt, and wt/wt mice (*P* < 0.001, for all three comparisons), and in all three* ob* genotypes on a 45% fat diet and for ob/wt and wt/wt on a 10% fat diet (*P* < 0.001, for all comparisons), but not for ob/ob mice on a 10% fat diet.

AUC was significantly higher in ob/ob mice compared with ob/wt and wt/wt mice based on all mice (*P* < 0.001) (Figure 1), in female mice (*P* < 0.001) and in male mice (*P* < 0.001) separately (*P* values similar for both genotype comparisons). The ob/wt mice had higher AUC than the wt/wt mice based on all mice (*P* = 0.003), in females (*P* = 0.050), but not in males, separately, in mice on a 10% fat diet (*P* = 0.040), but not in mice on a 45% fat diet, and not in mice treated with either 0.9% NaCl or PhIP, separately.

Mice fed a 45% fat diet were heavier than mice fed a 10% fat diet based on all mice (*P* < 0.001) (Figure 1), in both female and male ob/ob mice (*P* < 0.001, for both comparisons), and in male wt/wt mice (*P* = 0.005).

There were significant interactions between* ob* genotype and gender (*P* = 0.011),* ob* genotype and % fat diet (*P* ≤ 0.001) and* ob* genotype and treatment with 0.9% NaCl or PhIP (*P* = 0.027). There was a significant interaction between gender, % fat diet and* ob* genotype (*P* = 0.019), where the gender X % fat diet interaction depended on the* ob* genotype (*P* ≤ 0.001, for all three* ob* genotypes).

In addition to evaluating body weight as AUC, the terminal body weight and terminal BMI (data not shown) were also calculated for comparison. The body weight results were very much the same whether the data were evaluated as AUC from week 3 to 11 (Figure 1), terminal bw or terminal BMI at 11 weeks (data not shown), except that there was no difference in BMI between ob/wt and wt/wt mice. In addition, BMI was higher in ob/ob females than in males, whereas ob/ob males were larger than females for body weight as AUC and terminal body weight.

### 3.3. Small Intestinal Tumors in* Apc* ^*Min/+*^ Mice

**Figure 2:**
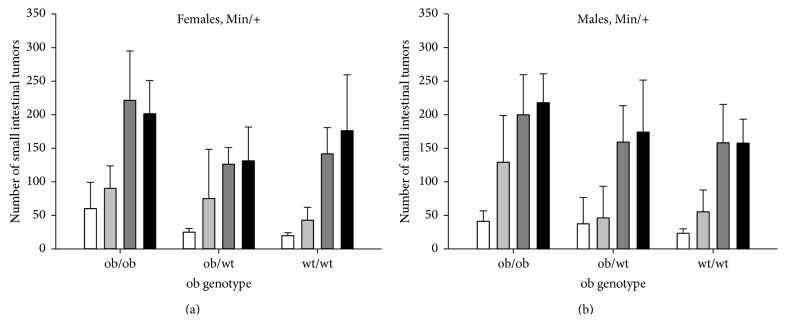
Number of small intestinal tumors. The number of small intestinal tumors (mean ± SD) is shown for (a) female and (b) male* Apc* ^*Min/+*^ mice with the three* ob* genotypes, terminated at 11 weeks of age. The mice were exposed to 0.9% NaCl and given a 10% fat diet (white columns) or 0.9% NaCl and a 45% fat diet (light grey columns), or they were exposed to PhIP and given a 10% fat diet (dark grey columns) or PhIP and a 45% fat diet (black columns). *n* = 10–17 mice.

All* Apc* ^*Min/+*^ mice (*n* = 10–17 in each experimental group), regardless of* ob* genotype, % fat diet, or 0.9% NaCl or PhIP treatment, had small intestinal tumors (adenomas), confirming 100% incidence of small intestinal tumors as is usually found in the* Apc* ^*Min/+*^ mice [15, 16, 19]. No tumors were found in the* Apc* ^*+/+*^ mice. The number of small intestinal tumors was not significantly different between female and male* Apc* ^*Min/+*^ mice (Figures 2(a) versus 2(b)). There were no significant interactions between gender and PhIP treatment, % fat diet or* ob* genotype for this end point.

The number of small intestinal tumors was significantly higher in ob/ob mice compared with ob/wt mice, based on all mice (*P* < 0.001), in PhIP-treated mice (*P* < 0.001) and in 0.9% NaCl-treated mice (*P* = 0.002), separately, and in ob/ob mice compared with wt/wt mice, for all mice (*P* < 0.001), in PhIP-treated mice (*P* < 0.001) and in 0.9% NaCl-treated mice (*P* < 0.001), separately (Figure 2). The number of small intestinal tumors was not significantly different in ob/wt mice compared with in wt/wt mice, based on all mice or in PhIP-treated or 0.9% NaCl-treated mice separately. There was a borderline significant interaction between* ob* genotype and PhIP treatment (*P* = 0.049).

The 45% fat diet increased the number of small intestinal tumors compared with the 10% fat diet, in all mice (*P* < 0.001), in vehicle-treated mice (*P* < 0.001), but not in PhIP-treated mice. There was a significant interaction between % fat diet and PhIP treatment (*P* = 0.007).

PhIP increased the number of small intestinal tumors compared with the vehicle 0.9% NaCl, in all mice (*P* < 0.001), in mice given a 10% fat diet (*P* < 0.001) and a 45% fat diet (*P* < 0.001). PhIP increased the number of small intestinal tumors in ob/ob mice (*P* < 0.001), in ob/wt mice (*P* < 0.001) and in wt/wt mice (*P* < 0.001), and in both females (*P* < 0.001, for all three comparisons) and males (*P* < 0.001, for all three comparisons) of each* ob* genotype (Figure 2).

**Figure 3:**
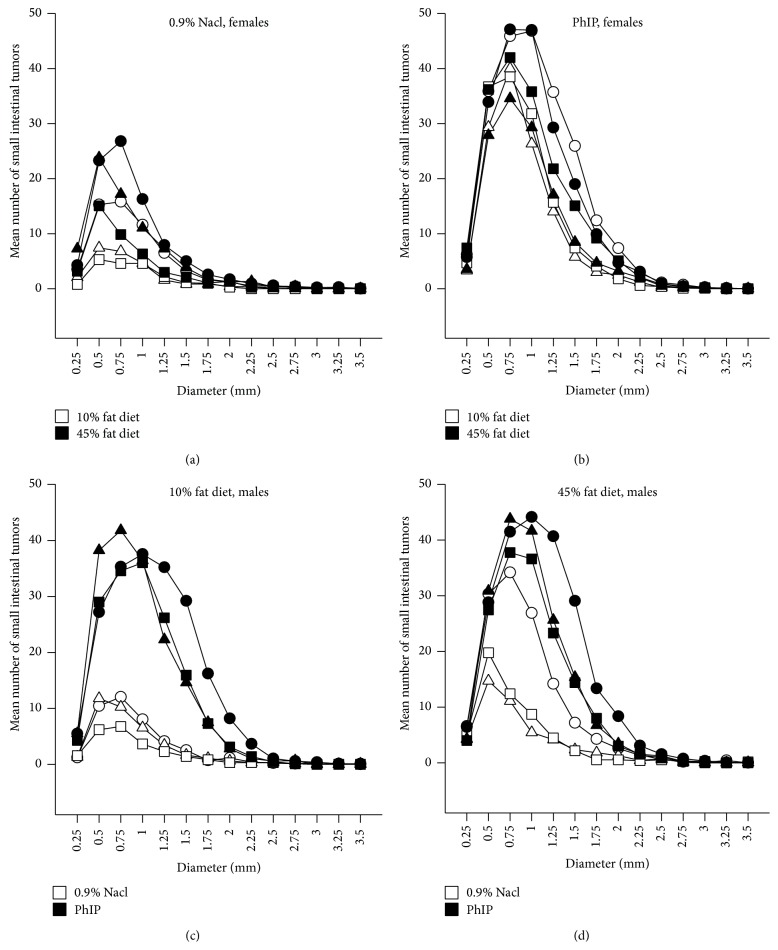
The size of the small intestinal tumors. This is illustrated by curves of distributions of tumors size classes (of 0.25 mm tumor diameter intervals) calculated as mean number of tumors in each tumor size class for each treatment group. The effect of a 45% fat diet compared with a 10% fat diet is shown for female* Apc* ^*Min/+*^ *X Lep* ^*ob/ob*^ mice (○),* Apc* ^*Min/+*^ *X Lep* ^*ob/wt*^ (∆) and* Apc* ^*Min/+*^ X* Lep* ^*wt/wt*^ mice (□) on a 10% fat diet, and the same genotypes on a 45% fat diet (filled symbols) exposed to (a) 0.9% NaCl, or (b) PhIP. The effect of exposure to PhIP compared with 0.9% NaCl is shown for male* Apc* ^*Min/+*^ *X Lep* ^*ob/ob*^ mice (○),* Apc* ^*Min/+*^ *X Lep* ^*ob/wt*^ (∆) and* Apc* ^*Min/+*^ X* Lep* ^*wt/wt*^ mice (□) exposed to 0.9% NaCl, or the same genotypes exposed to PhIP (filled symbols) on (c) a 10% fat diet, or (d) a 45% fat diet. *n* = 10–17 mice.

The small intestinal tumors had diameters of 0.15–4.30 mm. The statistical evaluation of differences in small intestinal tumor size between the experimental groups is described in the following (data not shown). In addition, this is illustrated with size distribution curves (Figure 3). Based on all mice, the small intestinal tumors were significantly larger in male mice compared with in female mice (*P* < 0.001). This was observed within all the PhIP-exposed mice (*P* < 0.001), also among the subgroups of PhIP-treated ob/ob mice (*P* < 0.001), ob/wt mice (*P* = 0.006) and wt/wt mice (*P* < 0.001), but not in 0.9% NaCl-treated mice. The males had larger tumors than the females also in the subgroup of ob/ob mice on a 10% fat diet (*P* < 0.001) and on a 45% fat diet (*P* < 0.001), in ob/wt mice on a 45% fat diet (*P* < 0.001), and in wt/wt mice on a 10% fat diet (*P* < 0.001).

The diameter of the small intestinal tumors was significantly higher in all ob/ob mice compared with ob/wt and wt/wt mice (*P* < 0.001, for both comparisons), but not in ob/wt compared with wt/wt mice (Figure 3, data not shown).

The effect of diet on the size of the small intestinal tumors was evaluated for both genders and is illustrated with size distribution curves for the females in Figures 3(a) and 3(b). The diameter of the small intestinal tumors was not significantly different between mice on a 10% fat diet versus on a 45% fat diet, based on all mice. Some statistically significant differences were observed in various subgroups, but these differences were not consistent in direction with regard to % fat diet and may therefore be due to chance. The tumors were not significantly larger after a 45% fat diet in the 0.9% NaCl-exposed or in the PhIP-exposed subgroups of mice. But this was the case in PhIP-exposed ob/wt and wt/wt mice (*P* < 0.001, for both comparisons), but not for the PhIP-exposed ob/ob mice. Both female and male ob/ob mice had significantly smaller tumors after a 45% fat diet compared with a 10% fat diet (*P* < 0.001, for both comparisons), possibly indicating formation of new tumors.

The effect of PhIP exposure on the size of the small intestinal tumors was evaluated for both genders and is illustrated with size distribution curves for the males in Figures 3(c) and 3(d). Based on all mice, the small intestinal tumors were significantly larger after PhIP exposure compared with 0.9% NaCl exposure (*P* < 0.001). This was also observed in females and males separately, and in mice on a 10% fat diet and a 45% fat diet separately (*P* < 0.001, for all comparisons). The small intestinal tumors were significantly larger after PhIP exposure compared with 0.9% NaCl exposure in all ob/ob mice (*P* < 0.001), in ob/wt mice on a 10% fat diet (*P* < 0.001) and a 45% fat diet (*P* < 0.001), and in wt/wt mice on 45% fat diet (*P* < 0.001). Within each gender separately, this was also the case for the subgroups of all three* ob* genotypes (*P* < 0.001, for all comparisons).

There were significant statistical interactions between % fat diet, gender and* ob* genotype (*P* ≤ 0.001), PhIP treatment, gender and* ob* genotype (*P* = 0.008), PhIP treatment, % fat diet and* ob* genotype (*P* ≤ 0.001), between PhIP treatment and gender (*P* = 0.009), and between PhIP treatment and % fat diet (*P* = 0.036), for this end point.

### 3.4. Colonic Tumors in* Apc* ^*Min/+*^ Mice

**Figure 4:**
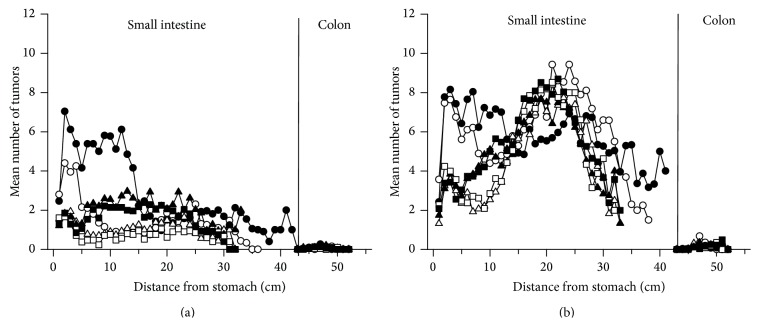
Localization of tumors along the small intestine and colon. This is shown for pooled female and male* Apc* ^*Min/+*^ *X Lep* ^*ob/ob*^ mice (○),* Apc* ^*Min/+*^ *X Lep* ^*ob/wt*^ (∆) and* Apc* ^*Min/+*^ X* Lep* ^*wt/wt*^ mice (□) on a 10% fat diet (open symbols) or a 45% fat diet (filled symbols) treated with (a) 0.9% NaCl or (b) PhIP. The tumor position is given as distance from the stomach measured in cm. Mean number of tumors/cm intestine for the mice in each experimental group was scored. *n* = 10–17 mice.

In the C57BL/6J strain, the small intestine is the main target organ for tumorigenesis caused by the* Min* mutation as well as by PhIP. Very few tumors were found in the colon in this experiment (Figure 4), adding no additional insight. Therefore, the data for incidence, number and size of the colonic tumors are not shown.

### 3.5. Localization of Tumors in the Small Intestine and Colon

In mice given the 10% fat diet, the majority of small intestinal tumors were localized in the distal two-thirds, that is, in middle and distal parts, of the small intestine (Figure 4), as is usually observed in the* Apc* ^*Min/+*^ mouse [15, 16, 19]. In the ob/ob mice, there were unusually high numbers of small intestinal tumors also in the proximal part, whereas in ob/wt and wt/wt mice there were much fewer tumors in this area. This distribution of tumors was observed both in 0.9% NaCl-treated (Figure 4(a)) and PhIP-treated (Figure 4(b)) mice, and after both treatments the tumor numbers were higher in this area with a 45% fat diet compared with a 10% fat diet. The few colonic tumors present were localized mainly in the middle to distal part of the colon.

### 3.6. Glucose Tolerance Test (GTT) and Nonfasted Blood Glucose

**Figure 5:**
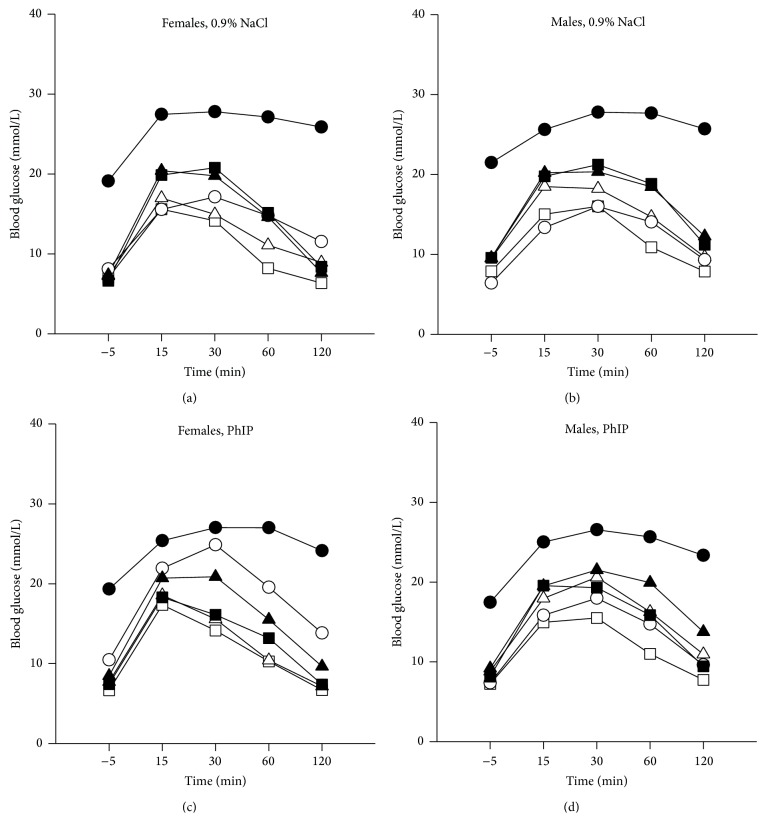
Blood glucose levels as area under the curve (AUC) from a glucose tolerance test (GTT). The GTT was performed on mice fasted for 6 h at 6 weeks of age. Blood glucose was measured 5 min before and 15, 30, 60 and 120 min after an i.p. injection of 2 g/kg bw glucose. The data were pooled for* Apc* ^*Min/+*^ and* Apc* ^*+/+*^ mice. The mice were treated with either 0.9% NaCl, (a) females and (b) males, or with PhIP, (c) females and (d) males. The mice had* ob* genotype ob/ob (○), ob/wt (∆) or wt/wt (□) and were given a 10% fat diet (open symbols) or a 45% fat diet (filled symbols). *n* = 4–9 mice.

Based on the AUC values from the GTT test performed at 6 weeks of age, AUC in* Apc* ^*Min/+*^ mice were higher than in* Apc* ^*+/+*^ mice, and AUC in PhIP-treated mice was higher than in 0.9% NaCl-treated mice; however, these comparisons did not reach statistical significance. The data for* Apc* ^*Min/+*^ and* Apc* ^*+/+*^ mice were therefore pooled in the curves of blood glucose levels after challenge (Figure 5). There were 5–9 mice in each experimental group, except 4 in one group; female wt/wt mice exposed to PhIP and a 45% fat diet. In GTT, there was no significant difference (n.s.) between the genders, but the general tendency based on all mice was higher levels in males than in females, differing between the subgroups with females higher than males in ob/ob mice (n.s.), and males higher than females in ob/wt (*P* = 0.002) and wt/wt mice (n.s.).

The ob/ob mice showed a much slower decrease in blood glucose than both the ob/wt and wt/wt mice (*P* < 0.001, for both comparisons) (Figure 5). This was also observed in females and males separately, and in mice on 45% fat diet (*P* < 0.001, for all comparisons). The ob/wt and wt/wt mice were not significantly different. In mice on a 10% fat diet, only the difference between ob/ob and wt/wt mice reached significance (*P* < 0.001). The male ob/wt mice had actually higher AUC than ob/ob mice both after treatment with 0.9% NaCl and PhIP on the 10% fat diet (Figures 5(b) and 5(d)).

Mice on a 45% fat diet had a slower blood glucose decrease after challenge than mice on a 10% fat diet (*P* < 0.001) (Figure 5). This was also observed in the subgroups ob/ob mice, ob/wt mice and wt/wt mice (*P* < 0.001, for all comparisons). Strikingly, the ob/ob mice on a 45% fat diet in particular had much higher and prolonged blood glucose levels after challenge, indicating severe blood glucose dysregulation in both genders (Figure 5).

There was a significant interaction between gender and* ob* genotype (*P* = 0.002) and between % fat diet and* ob* genotype (*P* < 0.001) in the GTT data.

According to WHO [20], diagnostic criteria for humans with impaired glucose tolerance (IGT) are 7.8–11.1 mmol/L 2 h after an oral GTT of 75 g glucose, and levels above 11.1 mmol/L confirm diabetes. Regarding these levels also relevant for mice, we found IGT at the 2 h time point in the GTT in 17% and 40%, 50% and 80%, 60% and 58%, 56% and 83%, 93% and 92%, and 100% and 82%, of the female and male mice, in the treatment groups wt/wt, ob/wt and ob/ob given a 10% fat diet, and wt/wt, ob/wt and ob/ob given a 45% fat diet, respectively. A diabetic level of glucose at the 2 h time point in the GTT was found in 0% and 0%, 20% and 20%, 20% and 50%, 0% and 33%, 21% and 67%, and 100% and 82%, of the female and male mice in the same treatment groups as above, respectively.

Nonfasted blood glucose levels were also measured in all mice at both age 6 (*n* = 10–18) and 11 (*n* = 9–17) weeks (data not shown). Based on all mice, there were higher blood glucose levels measured at week 6 compared with at week 11 (*P* < 0.001), but this difference varied between the subgroups. The* Apc* ^*Min/+*^ mice had significantly higher blood glucose levels than* Apc* ^*+/+*^ mice at 11 weeks (*P* < 0.001), but not at 6 weeks, confirming the GTT results. Based on all data, this was most pronounced in PhIP-exposed mice (*P* < 0.001), and in the ob/ob mice (*P* < 0.001). However, at 11 weeks separately, this difference between* Apc* genotypes was also found in the wt/wt mice (*P* = 0.038), but not in the ob/wt mice.

PhIP-treated mice had significantly higher blood glucose levels than 0.9% NaCl-treated mice (*P* < 0.001) at week 11, but not at week 6, confirming the GTT results. This was also observed in the subgroup* Apc* ^*Min/+*^ mice (*P* < 0.001), in mice given a 10% fat diet (*P* < 0.001) and in the ob/ob mice (*P* < 0.001), but not in the subgroup* Apc* ^*+/+*^ mice, in mice on a 45% fat diet or in ob/wt and wt/wt mice.

The nonfasted blood glucose data for comparisons between genders,* ob* genotypes and diets both at 6 and 11 weeks (data not shown) were essentially similar to the data from the GTT test.

### 3.7. Urine Glucose

In some mice showing high values of blood glucose, urine glucose was also measured by urinalysis dipsticks. These were mostly ob/ob mice, and 15 of the 23 (65%) ob/ob mice examined had glucose in their urine, whereas 8 of 23 (35%) had not. Among the ob/ob mice with glucose in the urine, all were females and mostly of* Apc* ^*Min/+*^ genotype and on 45% fat diet and had received either 0.9% NaCl or PhIP. However, also two examined female* Apc* ^*+/+*^ mice with wt/wt* ob* genotype on 45% fat diet exposed to 0.9% NaCl had glucose in their urine.

### 3.8. Insulin and IGF-1

**Figure 6:**
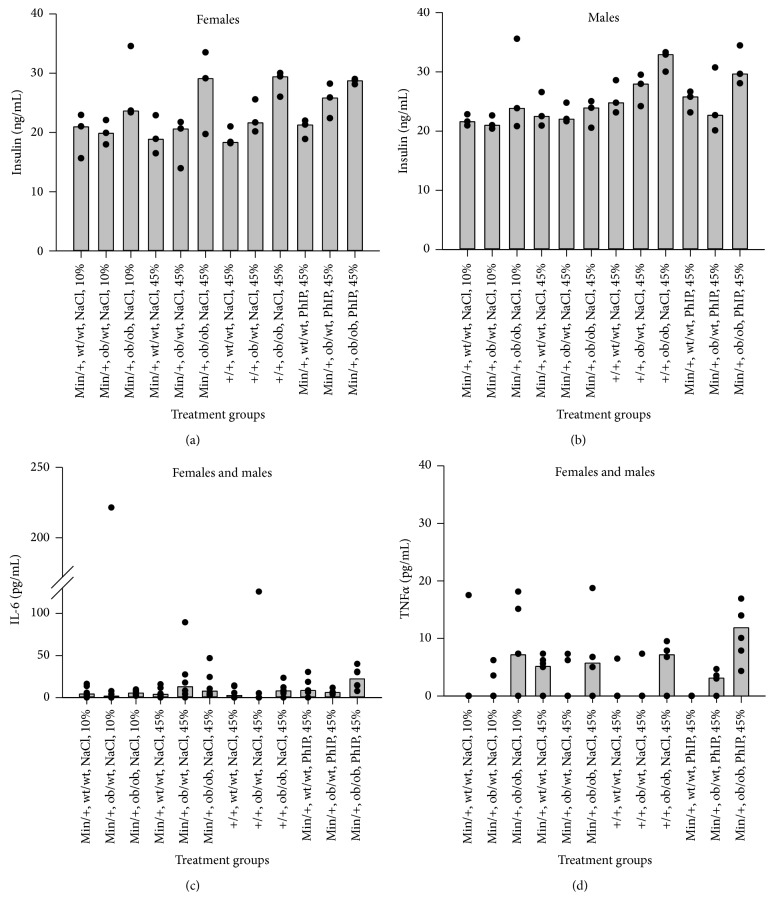
Levels of insulin and the proinflammatory cytokines IL-6 and TNF*α* in plasma. Insulin levels (ng/mL) were measured with ELISA in plasma obtained from the mice at termination, for (a) females and (b) males (median and individual values are shown as columns and dots, resp.). IL-6 (c) and TNF*α* (d) levels (both in pg/mL) were measured with bead-based immunoassays and analysed by flow cytometer in plasma obtained from the mice at termination. The data shown are from females and males combined (median and individual values are shown as columns and dots, resp.). *n* = 6 mice (3 of each gender).

Insulin and IGF-1 levels were measured in plasma obtained from the mice at termination. Six samples were analyzed from each experimental group with ANOVA, three from each gender. The levels of IGF-1 were below the limit of detection, 0.1 ng/mL, whereas insulin was detected in all samples (Figures 6(a) and 6(b)). The ob/ob mice had significantly higher levels of insulin than mice with ob/wt or wt/wt genotypes based on all mice (*P* < 0.001, for both comparisons), and also in the subgroups of both* Apc* genotypes, both genders, both diet groups and in 0.9% NaCl-treated mice (*P* values were <0.001–0.036). The ob/wt and wt/wt mice were not significantly different. In PhIP-treated mice, only the comparison between ob/ob and wt/wt reached significance (*P* = 0.012). The* Apc* ^*+/+*^ mice had higher insulin levels than* Apc* ^*Min/+*^ mice based on all mice (*P* = 0.028) and in the subgroup of males separately (*P* = 0.031). PhIP-treated mice had higher insulin levels than 0.9% NaCl-treated mice based on all mice (*P* = 0.026). Males (Figure 6(b)) had higher insulin levels than females (Figure 6(a)) based on all mice (*P* = 0.015), and in the subgroups of* Apc* ^*+/+*^ mice (*P* = 0.026) and* ob* wt/wt mice (*P* = 0.009). There were no significant differences in insulin levels between mice on a 10% or a 45% fat diet.

### 3.9. Cytokines

The proinflammatory cytokines IL-1*β*, IL-6 and TNF*α* were measured in plasma obtained from the mice at termination. Six samples were analyzed from each experimental group with ANOVA, three from each gender. The levels of IL-1*β* were below the limit of detection, 1.9 pg/mL, in all samples, whereas 78% and 46% of the samples had detectable levels of IL-6 and TNF*α*, respectively. No statistically significant differences were found in levels of IL-6 between any treatment groups (Figure 6(c)). Regarding TNF*α*, the ob/ob mice had significantly higher levels than mice with ob/wt or wt/wt genotypes based on all mice (*P* < 0.001, both comparisons), whereas ob/wt and wt/wt were not significantly different (Figure 6(d)). The significant difference in TNF*α* levels between mice with ob/ob genotype and ob/wt or wt/wt genotypes was seen in the mice given a 45% fat diet (*P* = 0.002, both comparisons), but not in the mice given a 10% fat diet. We found no significant differences in TNF*α* levels between mice exposed to 0.9% NaCl or PhIP (Figure 6(d)).

### 3.10. Correlations between Body Weight and Glucose, Insulin and Cytokine Levels

The strength of the association between the three different end points used for body weight and the other end points examined in this experiment was evaluated by simple linear regression (Table 2). The coefficient of determination (*R* ^2^) expresses the % of variation in the dependent variable than can be explained by the independent variables. The percentage of the variation in the nonfasted blood glucose levels measured at 6 weeks that could be explained by the body weight data was 46–57%, in the blood glucose levels measured at 11 weeks it was 33–43%, and in the insulin levels it was 32–48%, depending on which parameter was used for body weight (*P* < 0.001, for all comparisons). The percentage of the variation in the TNF*α* levels that could be explained by AUC and terminal body weight was around 20% (*P* = 0.021 and *P* = 0.013, resp.), whereas for BMI this association was not statistically significant.

### 3.11. Correlations between Body Weight, Glucose, Insulin and Cytokine Levels and Intestinal Tumorigenesis

The strength of the association between the body weight and the other end points examined in this experiment and number and diameter of small intestinal tumors was evaluated by simple linear regression (Table 3). Body weight evaluated as AUC from week 3 to 11 and nonfasted blood glucose levels at week 6 and week 11 could explain 11%, 12%, and 18% of the variation in the number of small intestinal tumors, respectively (*P* < 0.001, for all three variables). When evaluating number of small intestinal tumors with all three body weight parameters and blood glucose at both 6 and 11 weeks together with multiple linear regression, the association was statistically significant for body weight as AUC, terminal bw and blood glucose at 11 weeks (*P* < 0.001, for all three variables), explaining 31% of the variation in tumor number.

The association found by simple linear regression with diameter of small intestinal tumors and TNF*α*, body weight evaluated as AUC from week 3 to 11 and nonfasted blood glucose levels at week 6 and week 11, was 31%, 14%, 9% and 8%, respectively (*P* < 0.001, except for TNF*α* with *P* = 0.003) (Table 3). When evaluating diameter of small intestinal tumors with all three body weight parameters and blood glucose at both 6 and 11 weeks together with multiple linear regression, the association was statistically significant for body weight as AUC and terminal bw (*P* < 0.001, for both variables), explaining 21% of the variation in tumor diameter.

### 3.12. Long-Term Survival of Untreated ob Mice

**Figure 7:**
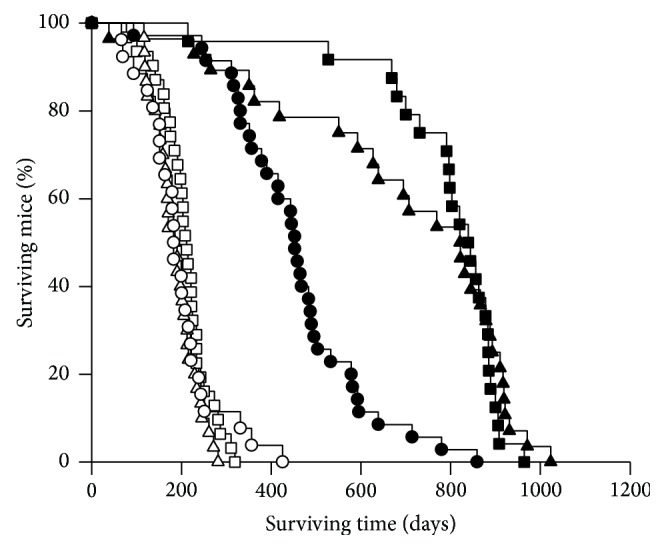
Survival of mice. Untreated pooled female and male* Apc* ^*Min/+*^ and* Apc* ^*+/+*^ mice of all three* ob* genotypes were kept under regular observation until euthanized. Survival of each genotype of mice was depicted as decreasing % of surviving mice compared with the number of mice present at the start of the experiment (*n* = 26, 30 and 31 for groups of* Apc* ^*Min/+*^ *X Lep* ^*ob/ob*^ (○),* Apc* ^*Min/+*^ *X Lep* ^*ob/wt*^ (∆) and* Apc* ^*Min/+*^ X* Lep* ^*wt/wt*^ (□) mice, respectively, and *n* = 35, 28 and 24 for* Apc* ^*+/+*^ *X Lep* ^*ob/ob*^ (●),* Apc* ^*+/+*^ *X Lep* ^*ob/wt*^ (▲) and* Apc* ^*+/+*^ *X Lep* ^*wt/wt*^ (■) mice, respectively.

For examination of impact of the* ob* genotype on life-span, groups of untreated* Apc* ^*Min/+*^ and* Apc* ^*+/+*^ mice of all three* ob* genotypes and both genders were kept under regular observation until euthanized (*n* = 24–35 per experimental group). Survival of each genotype of mice was depicted as decreasing % of surviving mice compared with the number of mice present at the start of the experiment (Figure 7). The life-span was significantly shorter for all three* Apc* ^*Min/+*^ X* ob* genotypes, because of their intestinal tumors causing anemia and other complications leading to early termination, compared with the* Apc* ^*+/+*^ X* ob* genotypes (*P* < 0.05, for all three comparisons). There were no significant differences between the three* ob* genotypes either of* Apc* ^*Min/+*^ or* Apc* ^*+/+*^ genotype when tested with one-way ANOVA. However, when tested with Student's *t*-test, or Mann-Whitney Rank sum test for data that failed the normality test, the* Apc* ^*+/+*^ ob/ob mice had significantly shorter life-span than the* Apc* ^*+/+*^ wt/wt and ob/wt mice (*P* ≤ 0.001, for both comparisons), whereas* Apc* ^*+/+*^ ob/wt and wt/wt mice were not significantly different.

## 4. Discussion

In this experiment, we have studied obesity as an end point in itself, and as a factor impacting on intestinal tumorigenesis. The obesity was either caused genetically, that is, by the inherited* ob* mutation in the* lep* gene, or caused by an environmental factor, a 45% fat diet, given from weaning to termination at 11 weeks of age. We have examined the effect of the obesity on both spontaneous intestinal tumors caused by the inherited* Min* mutation in the* Apc* gene, and on tumors induced by the mutagenic and carcinogen heterocyclic amine PhIP found in cooked meat and fish.

### 4.1. Genetically-Induced Obesity

In this experiment, we have evaluated obesity in three different ways: as body weight development using AUC calculated from weaning at 3 weeks to termination at 11 weeks of age, as terminal body weight and terminal BMI. This was done because various indicators of obesity, such as BMI, waist circumference and waist-to-hip ratio, waist-height-ratio or percentage of body fat, have been found to be more or less strongly associated with human study end points, and are therefore more or less suitable as indicators of obesity. All the three studied body weight parameters gave more or less the same differences between the experimental groups (Figure 1, data not shown), except that there was no difference in BMI between ob/wt and wt/wt mice. In addition, BMI was higher in ob/ob females than in males, whereas ob/ob males were larger than females for body weight as AUC and terminal body weight.

The mice with a homozygous* ob* mutation in the* lep* gene have a deficiency in the hormone leptin. These ob/ob mice were significantly heavier than the ob/wt and wt/wt, independent of body weight parameter (Figure 1, data not shown), reflecting the necessity of the leptin hormone for maintaining normal body weight. However, when we measured the feed intake for 24 h, the ob/ob mice actually had significantly lower intake of g feed or kcal/g bw than ob/wt and wt/wt mice (*P* < 0.001, for both comparisons), whereas ob/wt and wt/wt mice were not significantly different (Table 1). The ob/ob mice have been suggested to become obese because of hyperphagia [12]; however, our data indicate that they become obese despite of eating fewer grams of food and less calories per g body weight. According to information from the breeder [12], the ob/ob mice gain excess weight and deposit excess fat even when restricted to a diet sufficient for normal weight maintenance in lean mice. However, lower level of physical activity probably also contributes to the obesity in the ob/ob mice, since leptin is involved in regulation of energy expenditure [11, 13], and we observed that the ob/ob mice were much less physically active than the ob/wt and wt/wt mice.

### 4.2. Diet-Induced Obesity (DIO)

In addition to genetically-induced obesity caused by the* ob* mutation, we also studied the effects of diet-induced obesity (DIO), as an environmental factor, more relevant to the obese condition in most humans. The 45% fat diet significantly increased obesity, evaluated by all three body weight parameters, compared with the 10% fat control diet, based on all mice, in both genders, and in most subgroups of mice, except that the AUC for body weight was not significant in the subgroup of female wt/wt mice and in the ob/wt mice of both genders (Figure 1, data not shown).

DIO has been defined as a body weight of more than two standard deviations above the average body weight of mice fed a low-fat diet [21]. The ob/ob mice in all experimental groups, except the female* Apc* ^*Min/+*^ X ob/ob mice given 45% fat diet and PhIP, fulfilled this strict definition, based on terminal bw (data not shown). For* Apc* ^*Min/+*^ X ob/wt or wt/wt, of both genders, given 0.9% NaCl or PhIP, the mean in all eight groups was slightly below the body weight defining DIO, whereas for the* Apc* ^*+/+*^ X ob/wt or wt/wt, of both genders, given 0.9% NaCl or PhIP, 5 of 8 experimental groups fulfilled this definition. Except for the female and male* Apc* ^*+/+*^ X ob/wt given 0.9% NaCl and 45% fat diet, and the male* Apc* ^*+/+*^ X wt/wt given PhIP and 45% fat diet, slightly lower body weight can be explained by the fact that* Apc* ^*Min/+*^ mice generally have lower body weight than the* Apc* ^*+/+*^ because of their spontaneous intestinal tumors, affected even further with PhIP [19].

Mice seem to eat a constant amount of calories. Therefore, mice on a high fat diet would have a lower intake of food measured as g feed compared with mice on a low-fat diet. In this experiment, the mice fed a 45% fat diet had indeed significantly lower intake of g feed than mice fed the control 10% fat diet on a body weight basis (Table 1). It would be expected that the intake of the 45% fat diet was reduced until the same intake of calories was reached as with the 10% fat diet, but the intake of kcal/g bw/day was also significantly lower in the mice given the 45% fat diet (Table 1). Therefore, the observed difference in body weight between mice on a 10% fat and a 45% fat diet seems to be influenced also by the type of diet (i.e., a higher content of fat at expense of carbohydrates) and/or possibly reduced activity of the heavier mice on the 45% fat diet. The body weight increase was extreme in the ob/ob mice given the 45% fat diet, and their level of physical activity was noticeably reduced.

The ob/ob mice on a 45% fat diet also showed transient signs of diabetes, such as increased water intake and urine output, and glucose in the urine, as also described by the breeder [12]. However, there were no signs of the ob/ob mice being more hypothermic than the ob/wt and wt/wt mice, as described [12]. They seemed to thrive on regular temperature (20–24°C).

A recent paper describing a meta-review of micro-array data on high-fat DIO in C57BL/6J mice found that upregulated genes were associated with fatty acid synthesis, inflammation, signal transduction and transporters, and energy homeostasis [22]. Down-regulated genes were associated with sterol biosynthesis, insulin sensitivity and oxidative stress. Peroxisome proliferator activated receptor (PPAR)*γ* appeared to be a central obesity gene, interacting with lipid metabolism and inflammation genes.

### 4.3. Intestinal Tumorigenesis

In the* Apc* ^*Min/+*^ mice on the C57BL/6J background, the small intestine, rather than the colon, is the main target organ, and we therefore limit our discussion to the small intestinal tumors. In our study, homozygous mutation in the* ob* gene, causing deficiency in the hormone leptin in the ob/ob mice, significantly increased the number of spontaneous intestinal tumors and the number of PhIP-induced tumors in* Apc* ^*Min/+*^ mice compared with the mice having normal (wt/wt) or lower (ob/wt) levels of leptin (Figure 2). The tumor numbers were not significantly different between the ob/wt and wt/wt mice. The 45% fat diet further increased the number of small intestinal tumors based on all mice, and in the 0.9% NaCl-treated mice, that is, the spontaneous tumors. After PhIP exposure, the 45% fat diet did not increase the tumor numbers further based on all mice, and the response varied between the genotypes and genders (Figure 2).

Females and males had the same number of small intestinal tumors (Figure 2), but the tumors were significantly larger in males than in females, based on all mice (*P* < 0.001) and in many of the subgroups (data not shown), indicating a higher susceptibility in males to factors affecting tumor growth. Epidemiological evidence indicates a higher risk in men than in women for colorectal cancer [23, 24].

In the ob/ob mice, there were unusually high numbers of small intestinal tumors proximally in the small intestine, which were increased further with a 45% fat diet (Figure 4). It could be speculated whether this is caused by the increased secretion of bile acid into this small intestinal area caused by the high fat diet, as has been suggested to cause intestinal cancer [25].

The present data showed a consistent relationship between obesity (Figure 1, data not shown) and number of small intestinal tumors (Figure 2), both being increased in the ob/ob mice compared with the ob/wt and wt/wt mice, and the spontaneous tumors being further increased with the 45% fat diet. The same pattern was also observed in nonfasted blood glucose levels (data not shown). Body weight evaluated as AUC from week 3 to 11, and blood glucose levels at week 6 and week 11, showed the strongest association with number of small intestinal tumors with 11%, 12% and 18%, respectively (*P* < 0.001, all values) (Table 3).

In a previous study, similar results as in our study were found by crossing a mouse with a different mutation in the* Apc* gene (*Apc* ^*1638N/+*^) with the C57BLKS-*mLep* ^*db/db*^ mice, carrying a mutation in the leptin receptor gene (Ob-Rb) causing both obesity and diabetes mellitus [26]. At 6 months of age, these mice had increased numbers of tumors in their small intestine, and also tumors in the stomach, cecum, and colon, which were not seen in the* Apc* ^*1638N/+*^ mouse. In another study, the offspring from the crossing of the same* Min/+* mouse as we used (C57BL/6J-*Apc* ^*Min/+*^) and the C57BL/KsJ-*db/db* mouse, also having both obesity and diabetes, had increased number of total and small intestinal adenomas and increased incidence of colonic tumors at 15 months of age [27]. In these two studies, however, DIO was not included.

In humans, overweight and/or obesity are associated with increased risk, seen as increased incidence, mortality or poor prognosis for many types of cancer, including colon cancer [28–31]. Both body fatness and abdominal fatness have been evaluated as convincing increasing risks of colorectal cancer [32, 33].

### 4.4. Three Main Hypotheses for the Association between Obesity and Intestinal Tumorigenesis

There seem to be numerous signaling molecules and pathways through which obesity may impact on cancer, and considerable cross-talk and convergence between the pathways involved, ultimately ending in increased cell proliferation, cell growth or angiogenesis, or reduced apoptosis, and thus tumor promotion or progression. At least three main hypotheses, mutually interconnected, exist for the association between obesity and colorectal cancer [28–31, 34]: (1) obesity has been suggested to be associated with colon cancer via changes in adipokine levels released from the adipose tissues locally or systemically, such as increase in leptin and/or decrease in adiponectin, (2) that disturbed blood glucose regulation observed as increased serum levels of glucose, insulin and IGF-1 and insulin resistance lead to increased proliferation and reduced apoptosis, ultimately causing cancer, (3) or that obesity is a chronic low-grade inflammatory state in the adipose tissues releasing numerous proinflammatory cytokines, such as TNF*α*, IL-6 and IL-1*β*, affecting cancer. In addition, steroid hormones such as estrogen and androgen may be involved, but their role in colon cancer is controversial.

Regarding the* first hypothesis* of changes in adipokine levels as the link between obesity and colon cancer, we investigated the influence of leptin by the use of leptin-deficient mice. The diameter of the small intestinal tumors was significantly higher in all ob/ob mice compared with ob/wt and wt/wt mice (*P* < 0.001, for both comparisons) (Figure 3, data not shown); thus, the obesity increased the tumor size in spite of lack of growth-promoting leptin, in the ob/ob mice. As opposed to the ob/ob mouse, common obesity in humans is characterized not by lack of leptin, but rather by elevated levels of plasma leptin correlated with fat mass and BMI, representing a form of leptin resistance [35, 36].

However, the causative role of increased leptin in colon cancer is controversial and the data contradictory. Dysregulation of leptin or its receptors may contribute to colon cancer via effects on growth and proliferation of cancer cells via activation of signaling pathways, including JAK/STAT, PI3-kinase/AKT, and/or MAP kinases [37]. Leptin stimulated proliferation of human colon cancer cells* in vitro* and in rat colon [38] and promoted motility and invasiveness of human colon cancer cell lines [39], but had either potentiating or inhibiting effect on growth of human cancer cells from various other organs* in vitro* [40]. Leptin reduced the development of aberrant crypt foci (ACF), precancerous lesions in rat colon mucosa, induced by the carcinogen azoxymethane (AOM) [41], and leptin was found not to be a promotor in development of ACF in the colons of ob/ob and db/db mice [42]. Others found that leptin acts as a growth factor for colorectal tumors after initiation with AOM in mice [43].

In our experiment, the dietary fat is not affecting tumor growth as such, since the diameter of the small intestinal tumors was not significantly higher in mice on a 45% fat diet compared with the 10% fat diet, based on all mice. Some statistically significant differences were observed in the various subgroups, but the differences were not consistent in direction with regard to % fat diet. However, both female and male ob/ob mice had significantly smaller tumors after a 45% fat diet compared with a 10% fat diet (*P* < 0.001, for both comparisons), possibly indicating formation of new tumors (Figures 3(a) and 3(b), data not shown). It could be speculated that lipotoxic free fatty acids from the 45% fat diet [28, 44, 45], and/or the subsequent obesity [46], cause oxidative stress and accumulation of reactive oxygen species (ROS), which may lead to DNA damage and tumor initiation in the intestines. Genes involved in lipid metabolism, phospholipase A2, phospholipase Cɛ and phospholipase D, have been shown to modulate the intestinal tumor numbers by interacting with the Wnt signaling pathway [47–49]. However, controversy exists regarding the association between dietary animal fat as such and human colon cancer [50]. It may be that other factors than fat in the diet, such as heme iron in meat or carcinogens produced by cooking of meat, or the total energy, are also important for colon cancer.

The* second hypothesis* suggests that disturbed blood glucose regulation may lead to increased proliferation and reduced apoptosis, ultimately causing cancer. In our dynamic assessment of glucose regulation using GTT, ob/ob mice showed a much slower decrease in blood glucose than both ob/wt and wt/wt mice (*P* < 0.001, for both comparisons) (Figure 5). The nonfasted blood glucose levels were also significantly higher in ob/ob mice compared with both ob/wt and wt/wt mice at both 6 and 11 weeks (*P* < 0.001 for all comparisons) (data not shown), whereas there was no difference between ob/wt and wt/wt mice, following the same pattern as genetically induced-obesity.

Mice on a 45% fat diet had a slower blood glucose decrease after challenge in GTT than mice on a 10% fat diet (*P* < 0.001) (Figure 5). This was also observed in the subgroups ob/ob mice, ob/wt mice and wt/wt mice (*P* < 0.001, for all comparisons). The AUC in GTT was remarkably large in the ob/ob mice on a 45% fat diet (Figure 5), demonstrating a markedly reduced ability to regulate blood glucose. Nonfasted blood glucose levels were also significantly higher in all mice on a 45% fat diet compared with a 10% fat diet at both age 6 and 11 weeks (*P* < 0.001, for both comparisons) (data not shown).

The ob/ob mice had significantly higher levels of insulin than mice with ob/wt or wt/wt genotypes, based on all mice (*P* < 0.001, for both comparisons) (Figures 6(a) and 6(b)). The ob/wt and wt/wt mice were not significantly different. There were no significant differences in insulin levels between mice on a 10% or a 45% fat diet. However, the effect of the fat diets on insulin levels should be interpreted with caution because of the low number of samples, especially from mice on a 10% fat diet.

Overweight and obesity may cause secondary changes such as hyperglycemia, hyperinsulinemia and insulin resistance, and type 2 diabetes, which may lead to cancer development [34, 51, 52]. The hyperinsulinemia may enhance synthesis of insulin-like growth factor 1 (IGF-1) and its bioavailability by decreasing the IGF binding proteins (IGFBP) [53, 54]. It is thought that macrophage-related inflammatory activities in white adipose tissue (WAT) may contribute to obesity-induced insulin resistance [55, 56]. Leptin can affect insulin and glucose metabolism [57]. Studies have shown that both a lack of leptin signals and adiposity as such may contribute to insulin resistance and that decrease in central leptin signaling can critically affect glucose metabolism in obese mice [58].

An overall increase in glucose by age 20 weeks versus 6 weeks reported in the wild-type C57BL/6J mouse [59] was not clearly observed in our mice, since the glucose levels varied with the genotypes; significantly higher at week 11 versus week 6 in ob/wt mice (*P* = 0.017) or in the same direction but nonsignificant in* Apc* ^*Min/+*^ mice and wt/wt mice, whereas higher levels were seen at week 6 compared with week 11 in* Apc* ^*+/+*^ mice (*P* < 0.001) and in the ob/ob mice (*P* < 0.001). Hence, the genotypes, as well as the fat diets and/or the PhIP treatment, affected these results.

We observed a higher level of nonfasted blood glucose in male ob/wt and wt/wt mice compared with females both at age 6 and 11 weeks (data not shown). The same tendency was seen in the GTT. This gender difference is in accordance with reference values of blood glucose in the wild-type C57BL/6J mouse obtained both at 6 and 20 weeks of age [59]. However, as opposed to the normal wild-type mice, the female ob/ob mice had higher glucose levels than the males.

The* Apc* ^*Min/+*^ mice had significantly higher nonfasted blood glucose levels than* Apc* ^*+/+*^ mice at 11 weeks (*P* < 0.001) (data not shown). This was not caused by higher feed intake since there were no significant differences in feed intake between* Apc* ^*Min/+*^ and* Apc* ^*+/+*^ mice (Table 1), and the body weight was lower in* Apc* ^*Min/+*^ mice than* Apc* ^*+/+*^ mice for all three body weight scores (Figure 1, data not shown). A possible explanation is that APC is involved in regulation of epithelial glucose transport in the intestines, since* Apc* ^*Min/+*^ mice had increased activity of the electrogenic glucose carrier (SGLT1) compared with* Apc* ^*+/+*^ mice [60].* Apc* is a component of the Wnt signaling pathway [5, 6]. Therefore, other intriguing possibilities of a relationship between* Apc* and blood glucose levels come from data showing that the Wnt signaling pathway, which is as an important modulator of adipocyte differentiation [61, 62], also influences endocrine pancreas development and modulates mature *β*-cell functions including insulin secretion, survival and proliferation, and thereby may be involved in the pathogenesis of diabetes [63]. Components of the Wnt signaling pathway may also be involved in determining susceptibility to DIO [64].

The percentage of the variation in the blood glucose levels at 6 weeks that could be explained by the body weight data was 46–57%, and at 11 weeks it was 33–43%, depending on which parameter was used for body weight (*P* < 0.001, for all comparisons) (Table 2). The percentage of the variation in the insulin levels that could be explained by the body weight data was 32–48%, depending on which parameter was used for body weight (*P* < 0.001, for all comparisons) (Table 2). Body weight therefore seems to have a moderate effect on blood glucose regulation.

Overall, the increase in body weight (Figure 1) and the increased and prolonged blood glucose levels (Figure 5) correlate well with the increased number of small intestinal tumors (Figure 2).

In an epidemiological study, elevated insulin and glucose were associated with increased risk of colorectal adenoma and decreased apoptosis in normal rectal mucosa, indicating that insulin may act early in the adenoma-carcinoma sequence to promote the development of colorectal adenoma by decreasing apoptosis [65]. However, a diet high in foods that increased postprandial insulin levels did not increase the risk of colorectal cancer in a large prospective epidemiological study [66].

In the* third hypothesis*, inflammation has been suggested to be a causative link between obesity and colorectal cancer [28–31, 34]. There are two types of evidence indicating obesity as a chronic inflammatory illness: the release of proinflammatory cytokines such as TNF*α*, IL-6 and IL-1*β* from adipose tissues and infiltration of macrophages into the WAT [67]. In our study, we found a significantly higher level of TNF*α* in ob/ob mice compared with ob/wt and wt/wt mice, based on all mice, and in the subgroup of mice given a 45% fat diet, but not in the mice given a 10% fat diet (Figure 6(d)). The effect of the diets on TNF*α* should be interpreted with caution because of the low number of samples from mice on a 10% fat diet. The body weight data AUC and terminal body weight could explain around 20% of the variation in TNF*α* levels (*P* = 0.021 and *P* = 0.013, resp.) (Table 2). The variation in the number of small intestinal tumors could not be explained by the TNF*α* levels, whereas 31% of the variation in the diameter of small intestinal tumors could be explained by the TNF*α* levels (*P* = 0.003) (Table 3). Thus, our data support that inflammation is associated with obesity and the size of small intestinal tumors.

After diet-induced obesity (60% fat diet) in C57BL/6 mice, increased expression in the colon of TNF*α* was found, which instigated alterations in several components of the Wnt signaling pathway leading to tumor transformation [68]. In a model with tumors induced by AOM and dextran sulfate sodium (DSS) in lean (C57BL6/J) and ob/ob mice, it was shown that when pathways involving TNF*α* were inhibited, tumor numbers and proliferation and apoptosis profiles were returned to levels observed in lean mice [69]. By looking at the temporal sequence of inflammation in relation to tumorigenesis in* Min/+* mice, it was found that the increased mRNA expression of the inflammatory cytokines TNF*α*, IL-6, IL-1*β* and monocyte chemoattractant protein 1 (MCP-1) occurred at age 12 weeks in association with a rapid increase in tumor number at the same time, and that, in general, the overall tumor number and abundance of large tumors were positively correlated with the inflammatory cytokine responses [70]. Mice lacking TNF*α* because of a targeted null mutation were protected from insulin resistance caused by DIO or the ob/ob mutation [71]. However, a recent review and meta-analysis of epidemiological studies found no associations between colorectal cancer and TNF*α* and IL-6 [72].

We found no significant differences in TNF*α* levels between mice exposed to 0.9% NaCl or PhIP (Figure 6(d)). Also in rats, it was shown that inflammation did not precede or accompany the induction of preneoplastic lesions in the colon by PhIP [73].

### 4.5. Effects of Heterozygous ob Mutation

In our experiment, the ob/wt mice were significantly heavier than wt/wt mice when the body weight was evaluated as AUC (Figure 1) and as terminal bw (data not shown), but not as terminal BMI (data not shown). However, feed intake (Table 1), number (Figure 2) and size (Figure 3, data not shown) of small intestinal tumors, GTT results (Figure 5), nonfasted blood glucose levels (data not shown), insulin (Figures 6(a) and 6(b)) and TNF*α* (Figure 6(d)) levels were not significantly different between the ob/wt and wt/wt mice. Obviously, the heterozygous state of the* ob* mutation in mice was able to increase the body weight but was not severe enough to negatively affect other end points, including intestinal cancer.

Others have also shown that the ob/wt mice partially display the phenotype of the homozygous ob/ob mice, with approximately 33% reduction in plasma leptin level when adjusted for fat mass and 24% increase in percentage of body fat adjusted for age and sex [74]. The homozygous condition of ob/ob is found also in humans and is characterized by very low leptin levels and severe obesity [75]. This genetic condition is too rare to account for the recent obesity epidemic. It is more likely that most cases of common obesity in humans are caused by heterozygosity at several genetic loci for alleles with subtle effects on gene expression and function rather than by major functional disruption of any single gene. However, humans heterozygous for* ob* also have lower leptin levels and increased body fat percentage relative to wild-type relatives [76].

### 4.6. Survival

In our experiment, all* Apc* ^*Min/+*^ mice had reduced life-spans, due to anemia or other complications such as rectal prolapse, caused by the spontaneous intestinal tumors induced by the mutated* Apc* gene (Figure 7). Among these mice which have a limited life-span because of their tumors, the* ob/ob* genotype did not affect survival. However, in the* Apc* ^*+/+*^ mice being able to live out their natural life-span, the ob/ob mice had significantly shorter lives compared with ob/wt and wt/wt mice (Figure 7). The life-span was not significantly different between the* Apc* ^*+/+*^ ob/wt and wt/wt mice in our experiment, which was also reported by others, despite increased body weight and decreased leptin levels [74].

## 5. Conclusions

We have studied obesity induced both genetically via the* ob* mutation and via a 45% fat diet and shown that the high fat diet severely exacerbates the inherited obesity. In mice both with genetically-induced obesity and exposed to a 45% fat diet, spontaneous intestinal tumorigenesis was increased. The carcinogen- (PhIP-) induced intestinal tumorigenesis was not further increased by a 45% fat diet. Hyperglucosemia and insulinemia, indicating disturbed glucose regulation, and inflammation as seen by increased TNF*α* levels, were found associated with the obesity, implicating these as possible mechanisms involved in the association between obesity and intestinal tumorigenesis. The genetically-induced obesity decreased the life-span of the ob/ob mice.

## Figures and Tables

**Table 1:** Feed and water intake in mice kept in metabolic cages for 24 h.

*ob* genotype	% fat diet	Gender	*n*	Feed intake (g feed/g bw/day)	Feed intake (kcal AFE/g bw/day)	*n *	Water intake (mL/g bw/day)
wt/wt	10	F	10	0.25 ± 0.04	0.90 ± 0.13	10	0.25 ± 0.05
		M	11	0.18 ± 0.05	0.65 ± 0.19	11	0.20 ± 0.04

ob/wt	10	F	10	0.22 ± 0.03	0.82 ± 0.12	11	0.27 ± 0.06
		M	14	0.20 ± 0.05	0.72 ± 0.18	15	0.22 ± 0.06

ob/ob	10	F	11	0.17 ± 0.04	0.62 ± 0.14	11	0.31 ± 0.16
		M	8	0.16 ± 0.02	0.59 ± 0.06	9	0.23 ± 0.10

wt/wt	45	F	12	0.10 ± 0.02	0.44 ± 0.11	12	0.18 ± 0.04
		M	13	0.07 ± 0.03	0.30 ± 0.14	8	0.13 ± 0.04

ob/wt	45	F	12	0.09 ± 0.03	0.41 ± 0.14	11	0.15 ± 0.05
		M	14	0.07 ± 0.03	0.30 ± 0.13	10	0.12 ± 0.02

ob/ob	45	F	11	0.05 ± 0.02	0.25 ± 0.07	2	0.24 ± 0.08
		M	8	0.07 ± 0.02	0.31 ± 0.08	4	0.12 ± 0.01

Feed and water intake (mean ± SD) were recorded in some of the mice from each treatment group kept in metabolic cages for 24 h at age 9–11 weeks, after they were getting accustomed to the cages the day before. There were no consistent significant differences in feed and water intake between *Apc* ^*Min/+*^ and *Apc* ^+/+^ mice, or between 0.9% NaCl- or PhIP-treated mice, and therefore these data were pooled. F = females, M = males, and *n* = number of mice.

**Table 2:** Associations between various parameters for body weight and glucose, insulin and cytokine levels.

	AUC bw		Terminal bw		Terminal BMI	
	*R* ^2^	*P*	*R* ^2^	*P*	*R* ^2^	*P*
Terminal bw	0.91	<0.001				

Terminal BMI	0.59	<0.001	0.62	<0.001		

Glucose 6 weeks	0.46	<0.001	0.47	<0.001	0.57	<0.001

Glucose 11 weeks	0.33	<0.001	0.33	<0.001	0.43	<0.001

Insulin	0.34	<0.001	0.32	<0.001	0.48	<0.001

IL-1*β*	0.00	n.s.	0.00	n.s.	0.01	n.s.

TNF*α*	0.20	0.021	0.23	0.013	0.07	n.s.

Associations between various parameters for body weight (independent variables) and glucose, insulin, and cytokine levels (dependent variables) were examined with simple linear regression (SigmaPlot 12.3, Systat Software Inc., San Jose, CA, USA). This was performed on pairs of end points from all mice from all experimental groups from which individual data could be paired. Body weight (bw) data were evaluated either as area under the curve from week 3 to 11 (AUC bw), as terminal bw, or as terminal body mass index (BMI) at 11 weeks of age. Nonfasted blood glucose was measured at 6 and 11 weeks of age. Insulin was measured with ELISA, and TNF*α* was measured with flow cytometer, both in plasma obtained at termination at 11 weeks. *R* ^2^ = coefficient of determination, n.s. = not statistically significant.

**Table 3:** Associations between body weight, glucose, insulin and cytokine levels and the number or diameter of small intestinal tumors.

	Number of small intestinal tumors			Diameter of small intestinal tumors	
	*R* ^2^	*P*		*R* ^2^	*P*
Diameter of tumors	0.51	<0.001	No. of tumors	0.51	<0.001

AUC bw	0.11	<0.001	AUC bw	0.14	<0.001

Terminal bw	0.05	<0.001	Terminal bw	0.07	<0.001

Terminal BMI	0.08	<0.001	Terminal BMI	0.10	<0.001

Glucose 6 weeks	0.12	<0.001	Glucose 6 weeks	0.09	<0.001

Glucose 11 weeks	0.18	<0.001	Glucose 11 weeks	0.08	<0.001

Insulin	0.03	n.s.	Insulin	0.06	n.s.

IL-1*β*	0.00	n.s.	IL-1*β*	0.01	n.s.

TNF*α*	0.09	n.s.	TNF*α*	0.31	0.003

Associations between body weight parameters, glucose, insulin, and cytokine levels (independent variables) and the number or diameter of small intestinal tumors (dependent variables) were examined with simple linear regression (SigmaPlot 12.3, Systat Software Inc., San Jose, CA, USA). This was performed on pairs of end points from all mice from all experimental groups from which individual data could be paired. Number of small intestinal tumors is calculated as number of tumors in each mouse, and tumor diameter is calculated as mean of all tumors in each mouse. Body weight (bw) data were evaluated either as area under the curve from week 3 to 11 (AUC bw), as terminal bw, or as terminal body mass index (BMI) at 11 weeks of age. Nonfasted blood glucose was measured at 6 and 11 weeks of age. Insulin was measured with ELISA, and TNF*α* was measured with flow cytometer, both in plasma obtained at termination at 11 weeks. *R* ^2^ = coefficient of determination, n.s. = not statistically significant.
